# Complementary strategies to target residual immune activation and mucosal dysfunction in treated HIV: role of probiotics and pidotimod

**DOI:** 10.3389/fcimb.2026.1985254

**Published:** 2026-10-21

**Authors:** Katia Falasca, Raffaele Ferri, Jacopo Vecchiet

**Affiliations:** Clinic of Infectious Diseases, Department of Medicine and Science of Aging, G. d’Annunzio University of Chieti-Pescara, Chieti, Italy

**Keywords:** HIV, immune activation, immunostimulants, microbial translocation, microbiota, pidotimod, probiotics, recurrent respiratory infections

## Abstract

Alterations in the microbiota, loss of mucosal barrier integrity, and persistent immune activation are important determinants of susceptibility to infections and, in people with HIV receiving effective antiretroviral therapy, of non-AIDS comorbidities. This narrative mini-review examines two mechanistically distinct interventions within a shared translational hypothesis: strain-specific probiotics may act upstream by modifying the intestinal ecosystem and barrier function, whereas pidotimod may modulate downstream innate and mucosal immune responses. Evidence for probiotics in HIV remains heterogeneous across strains, formulations, doses, treatment duration, ART status, and endpoints. Pidotimod has shown immunological and clinical signals in small adult studies of community-acquired pneumonia and COVID-19, while HIV-specific evidence is currently limited to pilot study. These findings support further hypothesis-driven investigation but do not establish either a generalized probiotic class effect or pidotimod as a therapy for residual immune activation in treated HIV. Future randomized studies should separate validated clinical endpoints from exploratory biomarkers and evaluate safety, interactions, and patient subgroups prospectively.

## 1 Introduction

The relationship between the host and microorganisms cannot be reduced to a simple opposition between pathogens and the immune system. The microbiota contributes to colonization resistance, maturation of mucosal defenses, and the metabolism of numerous substrates; in parallel, the efficiency of innate and adaptive immunity influences the incidence, severity, and recurrence of infections. This perspective supports two complementary strategies: intervention on the microbial ecosystem through probiotics, prebiotics, or symbiotics, and direct modulation of the host response through immunostimulants. The term “probiotic” refers to live microorganisms that, when administered in adequate amounts, confer a health benefit on the host (Hill et al., 2014). This definition implies strain-level identification, a defined dose, viability, and demonstrated efficacy for a specific indication. The term “immunostimulant,” by contrast, encompasses highly diverse products—including bacterial lysates, synthetic compounds, and biologically derived immunomodulators—with non-equivalent mechanisms, manufacturing standards, and levels of evidence. This review critically examines the role of these interventions in infectious diseases, with particular attention to pidotimod and HIV.

Accordingly, this article is positioned as a hypothesis-generating perspective within a narrative mini-review, rather than as a comprehensive evaluation of all immunomodulatory options in treated HIV. Its purpose is to compare a specific dual-target proposal with selected lessons from the wider field and to define testable clinical questions, not to advocate routine use of either intervention.

### 1.1 Why probiotics and pidotimod are considered together

Probiotics and pidotimod are not members of a single pharmacological class and are not assumed to be interchangeable. The specific hypothesis of this review is that two different levels of the gut-immune axis may be addressed in parallel: selected probiotic strains may reduce an upstream driver of immune activation by improving microbial ecology, epithelial tight junctions, and microbial translocation, whereas pidotimod may influence downstream innate and mucosal response pathways. The proposed relationship is therefore complementary and translational, not evidence of a proven combined treatment effect.

In treated HIV, this framework is relevant because persistent mucosal damage and microbial translocation may coexist with dysregulated systemic immune activation. The review consequently asks whether interventions directed at the intestinal source and at host-response pathways generate biologically convergent signals. No clinical trial has yet established synergy, additivity, or clinical benefit from combining probiotics with pidotimod; this remains a testable hypothesis for future research (**Figure 1**).

**Figure 1:**
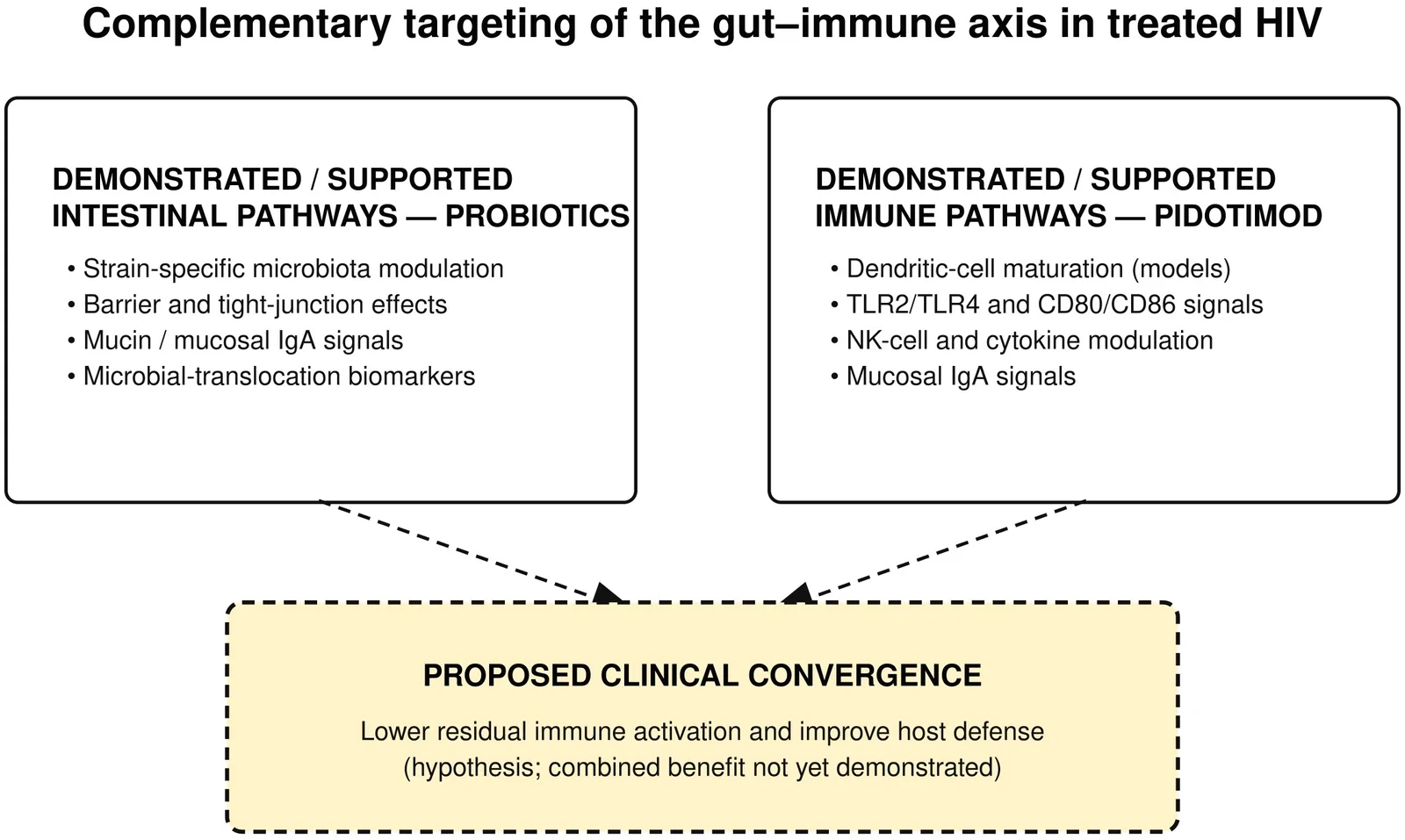
Solid boxes summarize mechanisms supported by experimental or human evidence; dashed arrows and the yellow box denote the proposed, not yet clinically demonstrated, convergence of probiotics and pidotimod on residual immune activation. ART, antiretroviral therapy; CD, cluster of differentiation; IgA, immunoglobulin A; NK, natural killer; TLR, Toll-like receptor.

## 2 Review methods

This narrative mini-review was informed by a focused PubMed/MEDLINE search covering January 1, 2014 to August 25, 2026. Earlier seminal studies were retained when required to define probiotic terminology, microbial translocation, trained immunity, or the historical pharmacology of pidotimod. Major NIH and European AIDS Clinical Society guidance and ClinicalTrials.gov records were also consulted. The principal search strings combined: (HIV OR people with HIV OR antiretroviral therapy) AND (probiotic* OR symbiotic* OR microbiota OR microbial translocation OR mucosal immunity); (pidotimod) AND (HIV OR pneumonia OR respiratory infection OR COVID-19); and (trained immunity) AND (TLR OR dendritic cell OR monocyte). Reference lists of key reviews were searched manually.

Eligible evidence included adult randomized or controlled trials, prospective or retrospective observational studies, pilot studies, systematic reviews and meta-analyses, mechanistic human or experimental investigations directly relevant to the proposed pathways, expert consensus documents, international guidelines, and registered adult trials. Exclusively pediatric studies, non-infectious indications without a relevant immune mechanism, reports lacking identifiable intervention characteristics, and publications not informative for the adult HIV-focused translational question were excluded. The restriction to adults reflects relevant differences between pediatric immune ontogeny and adult immunosenescence, comorbidity burden, chronic antigenic exposure, and ART-treated HIV.

The final narrative synthesis drew on 41 cited sources. Evidence was interpreted according to study design: systematic reviews and randomized trials were given greater weight when assessing efficacy; observational and pilot studies were considered hypothesis-generating; mechanistic investigations were used to assess biological plausibility; expert consensus documents reflected professional interpretation; and registered trials without peer-reviewed results were not considered evidence of efficacy. Consistent with the focused narrative design, no PRISMA flow diagram, formal risk-of-bias assessment, or quantitative meta-analysis was performed.

For reproducibility, the sources queried were PubMed/MEDLINE, ClinicalTrials.gov, the NIH ClinicalInfo guideline repository, and the European AIDS Clinical Society guideline website. The exact search interval was January 1, 2014 through August 25, 2026, with older seminal publications identified by backward citation searching. Core Boolean combinations were: (HIV OR people with HIV OR antiretroviral therapy) AND (probiotic* OR synbiotic* OR microbiota OR microbial translocation OR mucosal immunity); pidotimod AND (HIV OR pneumonia OR respiratory infection OR COVID-19); and (treated HIV OR antiretroviral therapy) AND (statin* OR chloroquine OR hydroxychloroquine OR IL-21 OR vedolizumab OR canakinumab OR IL-1 antagon*).

## 3 Probiotics in infectious diseases

### 3.1 Biological rationale and strain specificity

Probiotics may compete with pathogens for nutrients and adhesion sites, produce organic acids and bacteriocins, strengthen tight junctions, stimulate mucin and IgA production, and modulate dendritic cells, regulatory T lymphocytes, and Toll-like receptor-mediated signaling (Hill et al., 2014). These effects are formulation- and strain-dependent. Efficacy must therefore be attributed only to the specific microorganism(s), dose, matrix, duration, storage conditions, population, and endpoint investigated, rather than extrapolated to probiotics as a class (Hill et al., 2014; Su et al., 2020; Goodman et al., 2021).

### 3.2 Gastrointestinal infections and complications

The setting with the most consistent clinical signal is the prevention of antibiotic-associated diarrhea. A meta-analysis of 42 randomized studies in adults found a reduction in risk, with efficacy influenced by baseline risk, dose, and product composition (Goodman et al., 2021). However, this finding does not support a class effect: different guidelines reach different conclusions and endorse only selected combinations. For the prevention of Clostridioides difficile infection, the American Gastroenterological Association allows the use of specific formulations in patients receiving antibiotics, whereas other societies are more restrictive (Su et al., 2020). This discrepancy reflects study heterogeneity, relatively uncommon events, and uncertainty regarding generalizability. In acute gastroenteritis, efficacy varies according to age, geographical setting, and the strain used. Differences among trials confirm the need to select evidence-supported formulations and to define the expected outcome *a priori*, avoiding the assumption that all probiotics are equivalent (Hill et al., 2014; Su et al., 2020).

### 3.3 Respiratory infections

Systematic reviews suggest that certain probiotics may reduce the number or duration of acute upper respiratory tract infections (Hao et al., 2015). Despite heterogeneity in populations and products, the preventive signal is consistent with the ability of probiotics to modulate mucosal defenses. These products should be regarded as complementary to, rather than substitutes for, vaccination, hygiene, etiological diagnosis, and appropriate antimicrobial therapy.

### 3.4 Safety

Probiotics are generally well tolerated, but safety in people with HIV should be assessed in relation to immune and barrier status rather than inferred from healthy populations. Particular caution is appropriate in advanced or uncontrolled HIV, very low CD4 counts, severe mucosal barrier impairment, critical illness, central venous catheterization, or concurrent broad-spectrum antibiotic exposure. Rare Lactobacillus bacteremia and Saccharomyces fungemia have been reported, and antimicrobial-resistance determinants may be carried or transferred by inadequately characterized products (Kullar et al., 2023).

Risk assessment should include CD4 count and virological control, recent opportunistic infection, gastrointestinal pathology, intravascular devices, concomitant antimicrobials, and the exact product used. Preparations should provide strain-level identity, lot traceability, viability and purity testing, absence of clinically relevant transferable resistance determinants, and manufacturing quality control. Products studied in one formulation should not be substituted automatically with commercial preparations that have different strain composition or quality standards.

### 3.5 Next-generation probiotics and live biotherapeutic products

Akkermansia muciniphila and other next-generation probiotics differ from conventional food-associated probiotics because they may be developed as live biotherapeutic products (LBPs) with a defined strain, manufacturing process, potency specification, and intended disease indication. This regulatory category requires pharmaceutical-grade identity, purity, stability, absence of adventitious agents and transferable antimicrobial-resistance determinants, and clinically appropriate containment and shedding assessment (US Food and Drug Administration, 2016). Pasteurized or non-viable preparations may reduce some infection risks but cannot be assumed to reproduce the effects of a live strain.

In people with residual immune impairment, live-organism administration warrants particular caution regarding translocation, bacteremia or fungemia, altered susceptibility under antibiotic pressure, and product contamination. Advanced immunosuppression, severe barrier injury, central venous access, critical illness, and uncontrolled viremia should be prospectively addressed in eligibility and monitoring plans. No Akkermansia-based LBP currently has established efficacy for residual immune activation in treated HIV.

## 4 Immunostimulants in infectious diseases

The intended objective of host-directed immunomodulation is not indiscriminate immune stimulation. In chronic HIV, three goals must be distinguished: immune restoration (reconstitution of deficient cellular or mucosal compartments), reduction of pathological immune activation, and enhancement of host defense against infection. These outcomes can diverge, and excessive or poorly targeted stimulation could theoretically amplify inflammation. Accordingly, bacterial lysates and synthetic compounds should be evaluated according to their specific pathways, populations, and endpoints rather than grouped as uniformly beneficial immunostimulants (Giagulli et al., 2009; Puggioni et al., 2019; Zhao et al., 2019; Licari et al., 2024; Zygadło et al., 2026).

In adults, clinical interest has focused on recurrent respiratory infections, community-acquired pneumonia, and viral respiratory infections. Systematic-review, controlled-trial, observational, and mechanistic findings carry different evidentiary weight. Current data justify further evaluation of selected agents as adjuncts to established preventive or therapeutic measures, but do not support routine use across broadly defined immunocompromised populations (Trabattoni et al., 2017; Puggioni et al., 2019; Zhao et al., 2019; Santus et al., 2021; Ucciferri et al., 2022; Licari et al., 2024; Zygadło et al., 2026).

## 5 Pidotimod

### 5.1 Pharmacology and mechanisms

Pidotimod is a synthetic dipeptide developed for the prevention and adjunctive treatment of respiratory infections. Experimental and translational studies report dendritic-cell maturation, increased CD80/CD86 expression, modulation of TLR-related signaling, effects on natural killer-cell activity, and support of mucosal responses (Giagulli et al., 2009; Puggioni et al., 2019; Zhao et al., 2019; Licari et al., 2024). These findings should be separated from clinical evidence: dendritic-cell and nasal-mucosal effects derive principally from experimental or translational models, whereas human studies have mainly measured circulating or mucosal biomarkers alongside clinical outcomes in small cohorts. The description of pidotimod as a physiological immunomodulator is therefore best regarded as a proposed pharmacological characterization, not a clinically validated distinction from immunostimulation.

Trained immunity is mentioned here only as a theoretical framework for future research, not as a demonstrated pidotimod mechanism. Pidotimod has not been shown to induce the epigenomic, transcriptomic, metabolic, or functional-rechallenge changes required to establish trained innate immune memory (Netea et al., 2011; Kleinnijenhuis et al., 2012; Saeed et al., 2014; Netea et al., 2020).

### 5.2 Evidence in respiratory infections

Adult evidence for pidotimod includes small controlled studies, observational cohorts, mechanistic investigations, narrative reappraisals, and expert consensus in community-acquired pneumonia, COVID-19, and chronic respiratory disease (Giagulli et al., 2009; Trabattoni et al., 2017; Puggioni et al., 2019; Zhao et al., 2019; Santus et al., 2021; Ucciferri et al., 2022; Licari et al., 2024; Marseglia et al., 2025; Ucciferri et al., 2025). These sources provide a coherent biological rationale and some favorable signals, but their interpretation is limited by small samples, heterogeneous populations and treatments, and the absence of large independently replicated randomized trials. They therefore support further study of a possible adjunctive role rather than establish efficacy.

The 2024 reappraisal summarizes more than 30 years of pharmacological and clinical experience (Licari et al., 2024), while an Italian multidisciplinary Delphi consensus reports expert agreement on selected clinical uses (Marseglia et al., 2025). Such syntheses are valuable for contextualization but should not be assigned the same evidentiary weight as adequately powered randomized trials. The 2025 review by Ucciferri et al. similarly maps emerging applications and research priorities rather than providing independent confirmatory efficacy evidence (Ucciferri et al., 2025).

### 5.3 Community-acquired pneumonia and COVID-19 in adults

In community-acquired pneumonia, Trabattoni et al. randomized 16 hospitalized adults to levofloxacin alone or levofloxacin plus pidotimod 800 mg twice daily (Trabattoni et al., 2017). Combination therapy was associated with changes in antimicrobial and immunomodulatory proteins, TLR2/TLR4 and CD80/CD86 expression, and TNF-α-producing cells. The randomized design is a strength, but the very small sample and biomarker-focused outcomes preclude firm conclusions regarding clinical efficacy.

In mild-to-moderate COVID-19, an Italian non-randomized study included 184 adults; 97 received pidotimod and 87 received other treatments (Ucciferri et al., 2022). Emergency-department attendance or hospitalization, disease duration, and rescue corticosteroid use favored the pidotimod group. However, treatment allocation, evolving standards of care, heterogeneity of concomitant therapy, and residual confounding limit causal inference. Hospitalization and corticosteroid use may also be influenced by clinical practice and cannot alone establish antiviral efficacy.

A further study of 16 adults hospitalized with COVID-19 pneumonia reported a lower neutrophil-to-lymphocyte ratio, higher IFN-gamma, and increased Toll-like-receptor expression with pidotimod added to standard care (Santus et al., 2021). These are exploratory inflammatory and mechanistic biomarkers; the small sample, heterogeneous COVID-19 treatment context, and lack of validated clinical endpoints require cautious interpretation. Collectively, the COVID-19 studies generate a signal for further controlled investigation rather than demonstrate treatment effectiveness.

### 5.4 Tolerability

Pidotimod has generally been well tolerated in the available adult studies, with mostly mild and transient adverse events (Puggioni et al., 2019; Zhao et al., 2019; Santus et al., 2021; Ucciferri et al., 2022; Licari et al., 2024). Nevertheless, the small size and limited follow-up of several studies restrict detection of uncommon events. Published HIV data have not identified clinically relevant ART interactions (Ucciferri et al., 2021), but formal interaction studies are limited. Future protocols should prospectively assess ART regimen, vaccination timing, antibiotic exposure, corticosteroids, and other immunomodulatory or preventive interventions. Similar attention is required for probiotics because antibiotics may alter viability and ecological effects, while strain-specific immune effects could modify vaccine responses.

## 6 HIV: microbiota, immune activation, and complementary interventions

### 6.1 Rationale in treated HIV

Suppressive antiretroviral therapy markedly reduces morbidity and mortality but may not fully restore immune homeostasis. Gut-associated lymphoid-tissue damage, dysbiosis, impaired epithelial integrity, microbial translocation, coinfections, and viral persistence contribute to sustained immune activation (Brenchley et al., 2006; Hunt et al., 2014). IL-6, sCD14, D-dimer, and hsCRP are associated with non-AIDS events (Kuller et al., 2008; Tenorio et al., 2014), but reduction of these biomarkers is not itself a validated therapeutic endpoint. Current guidelines do not recommend routine biomarker-guided immunomodulatory treatment because lowering a marker has not been shown to improve clinical outcomes (US Department of Health and Human Services, 2025).

### 6.2 Probiotics in people with HIV

The probiotic evidence in HIV is heterogeneous across strains and combinations, doses, treatment duration, ART status, baseline immune recovery, and endpoints. A systematic review reported possible signals for diarrhea, CD4 counts, inflammation, and microbial translocation, but emphasized heterogeneity and limited certainty (Carter et al., 2016). Falasca et al. reported cytokine modulation in a preliminary study (Falasca et al., 2015), whereas the randomized VISBIOME ES trial documented microbiome changes during ART (Presti et al., 2021). These findings are formulation-specific and should not be generalized to probiotics as a class.

In 30 virologically suppressed adults, 18 received Vivomixx (1.8 × 10^12^ CFU/day) for four weeks before and after PCV13 vaccination and 12 received placebo (Reale et al., 2021). The formulation was associated with greater anti-pneumococcal CPS13 IgG, salivary IgA, IL-10, and IL-8 responses. This small study suggests an effect on vaccine-response biomarkers but was not designed to demonstrate fewer pneumococcal infections. Related work characterized cytokine and vascular-inflammatory mediators, including CD40 ligand, Dickkopf-1, and P-selectin, providing mechanistic context rather than evidence of clinical benefit (Falasca et al., 2015; Falasca et al., 2019).

In the RECOVER randomized trial of immunologically discordant adults, a synbiotic containing two Lactiplantibacillus plantarum strains and Pediococcus acidilactici produced small changes in the CD4/CD8 ratio and sCD14, whereas the probiotic-only arm did not significantly alter immune parameters or fecal microbiome composition (Blázquez-Bondia et al., 2022). Registered studies of Rillus and Lactobacillus casei Shirota (Yakult) address immune non-response, Th17 recovery, translocation, and NK-cell function, but registry entries without peer-reviewed results are ongoing or descriptive evidence, not proof of efficacy (Nelwan, 2018; University of São Paulo General Hospital, 2014).

Lactobacillus abundance has been linked in experimental lentiviral models to IDO1 activity and mucosal Th17 dynamics, selected Bifidobacterium strains may influence Treg/Th17 balance, and Akkermansia muciniphila is associated with mucus-layer ecology (Zevin et al., 2016). These mechanistic or ecological observations cannot be assumed to predict clinical effects in treated HIV. **Table 1** therefore compares the principal human HIV studies by formulation, design, population, exposure, and endpoint.

**Table 1:** Comparative summary of the principal probiotic and pidotimod clinical evidence discussed in this review.

Study/design	Population/n	Intervention and dose	Duration	Endpoints	Key findings/interpretation
Falasca et al. (2015); single-center open-label prospective study	Virologically suppressed men receiving ART; n=30	Lacticaseibacillus casei Shirota; Yakult Light, ≥6.5 × 10^9^ CFU/bottle, 1 bottle twice daily	4 weeks	Lymphocyte subsets, cytokines, cystatin C and microalbuminuria	Exploratory within-participant biomarker changes; no control arm or clinical endpoint
Presti et al. (2021); phase II randomized double-blind placebo-controlled trial	People with HIV receiving ART; n=93	VISBIOME ES multistrain formulation (approximately 1.8 × 10^12^ CFU/day) vs placebo	24 weeks	sCD14, inflammatory markers, gut permeability and microbiome	Safe and microbiome-active; no significant effect on systemic inflammation or gut permeability
Reale et al. (2021); placebo-controlled vaccine-response study	Virologically suppressed adults; n=30	Vivomixx, 1.8 × 10^12^ CFU/day vs placebo	4 weeks before and 4 weeks after PCV13	PCV13 IgG, salivary IgA, IL-10 and IL-8	Vaccine-response biomarkers; underpowered for clinical infections
Blazquez-Bondia et al (Blázquez-Bondia et al., 2022); double-blind placebo-controlled RECOVER trial	Immunologically discordant adults; n=89 randomized, n=71 analyzed	L. plantarum CECT7484/CECT7485 plus P. acidilactici CECT7483, with or without a fiber prebiotic; daily sachet	6 months plus 3-month washout	Safety, CD4/CD8 ratio, sCD14, inflammation and microbiome	Small synbiotic-arm signals of uncertain clinical significance; probiotic-only arm showed no immune effect
PIONIR/Rillus (Nelwan, 2018); randomized double-blind phase II registered trial	Immunological non-responders; n=80	L. plantarum, S. thermophilus and B. bifidum, 1.0 × 10^9^ CFU plus FOS; 1 chewable tablet nightly	12 weeks	CD4, Th17, blood 16S rRNA, fecal calprotectin and safety	Completed registry with posted results; no peer-reviewed primary outcome publication identified
Yakult trial (University of São Paulo General Hospital, 2014); randomized triple-masked registered trial	People with suppressed HIV and poor CD4 recovery; anticipated n=48	L. casei Shirota fermented milk, 4.0 × 10^10^ organisms/80 g once daily	12 weeks	CD4 recovery, immune activation, microbial translocation, NK function and microbiome	Completed registry; no peer-reviewed primary outcome publication identified
Trabattoni et al., 2017 (Trabattoni et al., 2017); randomized mechanistic trial	Adults hospitalized with CAP; n=16	Pidotimod 800 mg twice daily plus levofloxacin vs levofloxacin	During acute treatment	TLR2/4, CD80/86, TNF-alpha and immune proteins	Biomarker modulation; far too small for clinical efficacy
Ucciferri et al., 2022 (Ucciferri et al., 2022); non-randomized	Adults with mild-moderate COVID-19; n=184	Pidotimod 800 mg twice daily vs other care	7–10 days	Hospital/ED use, duration, rescue corticosteroids	Favorable associations; confounding and treatment heterogeneity
Santus et al., 2021 (Santus et al., 2021); small controlled study	Hospitalized COVID-19 pneumonia; n=16	Pidotimod plus standard care vs standard care	Short term	NLR, IFN-gamma, TLR expression	Exploratory biomarkers; no validated clinical endpoint
Ucciferri et al., 2021 (Ucciferri et al., 2021); controlled pilot	Virologically suppressed people with HIV; n=40 (30/10)	Pidotimod vs control	4 weeks plus 4-week follow-up	Cytokines, salivary IgA, cystatin C, microalbuminuria	Hypothesis-generating; imbalanced groups and no hard endpoint

### 6.3 Pilot study of pidotimod in HIV

The 2021 pilot study enrolled 40 people with HIV receiving ART: 30 received pidotimod and 10 served as controls (Ucciferri et al., 2021). Serial assessments included inflammatory, mucosal, cytokine, and renal biomarkers. Pidotimod exposure was associated with cytokine-profile changes, a sustained increase in salivary IgA, and reductions in cystatin C and microalbuminuria. These renal-marker changes indicate biological signals only; they do not demonstrate improved glomerular filtration, prevention of chronic kidney disease, or another clinically meaningful renal benefit.

The study was exploratory and is limited by its small sample, 3:1 group imbalance, restricted statistical power, biomarker multiplicity, and lack of independent replication. It was not powered for infections, non-AIDS events, immune recovery, or renal clinical outcomes. The findings should therefore be considered hypothesis-generating and used to inform endpoint selection and sample-size calculation in future randomized trials.

### 6.4 Positioning within the wider HIV immunomodulatory landscape

The proposed strategy should be viewed alongside prior attempts to modify residual inflammation in HIV. Chloroquine or hydroxychloroquine produced biomarker changes in small studies, but randomized evidence did not establish clinical benefit and, outside suppressive ART, hydroxychloroquine adversely affected CD4 counts and HIV RNA (Paton et al., 2012). IL-21, alone or combined with probiotics, improved intestinal Th17 preservation and microbial-translocation measures in ART-treated SIV models, but this remains preclinical for treated human HIV (Ortiz et al., 2016). Vedolizumab targets α4β7-dependent gut lymphocyte trafficking and has provided mechanistic information on reservoir biology, without establishing routine clinical benefit (Jimenez-Leon et al., 2024). IL-1β antagonism with canakinumab altered inflammatory or vascular signals in small treated-HIV studies, but has not demonstrated fewer non-AIDS events in this population (Hsue et al., 2018).

Statins provide the most instructive comparator. REPRIEVE demonstrated that pitavastatin reduced major adverse cardiovascular events in people with HIV at low-to-moderate predicted cardiovascular risk, thereby linking an intervention to a hard clinical endpoint rather than biomarker movement alone (Grinspoon et al., 2023). Pidotimod differs in its oral administration, generally favorable short-term tolerability, and putative mucosal/innate targets, but lacks phase III or independently replicated HIV evidence. These features justify comparative investigation, not preferential positioning.

### 6.5 The biomarker-to-clinical endpoint gap

The central translational limitation is the gap between movement in surrogate biomarkers and patient benefit. sCD14, IL-6, salivary IgA, cystatin C, and microalbuminuria may indicate pathway engagement or physiological change, but none establishes fewer cardiovascular events, malignancies, serious infections, or deaths. The HIV immunomodulation literature contains multiple examples in which inflammatory or mucosal markers changed without validated clinical benefit. Associations with prognosis do not make treatment-induced changes valid surrogate endpoints. The mechanistic and evidentiary differences between probiotics and pidotimod are summarized in **Table 2**.

**Table 2:** Mechanistic and evidentiary comparison of probiotics and pidotimod.

Domain	Probiotics	Pidotimod
Primary target	Strain-specific microbiota, epithelial barrier, microbial metabolites	Host innate/adaptive and mucosal immune pathways
Mechanisms supported	Tight junctions, mucin/IgA, ecological and immune signaling effects	Dendritic-cell maturation and TLR/costimulatory signals mainly in experimental/translational studies; human biomarker signals
HIV evidence	Heterogeneous randomized, controlled, pilot, and registered studies	Single 40-participant controlled pilot study
Outcomes	Microbiome composition, translocation/inflammation markers, CD4/CD8, vaccine-response markers	Cytokines, salivary IgA, cystatin C, microalbuminuria; no validated clinical HIV outcome
Key limitations	Strain/formulation heterogeneity; small samples; variable endpoints	Small samples, group imbalance, biomarker multiplicity, no independent HIV replication
Safety	Consider CD4 count, barrier injury, catheter, bacteremia/fungemia, AMR, product quality	Generally favorable short-term tolerability; uncommon events and interactions incompletely characterized
Status in treated HIV	Not recommended as a class for residual activation	Investigational; not guideline-recommended for residual activation

The absence of phase III evidence demonstrating a reduction in non-AIDS clinical events is the principal reason that current NIH and EACS guidance does not recommend routine immunomodulator use for residual immune activation. This is distinct from interventions such as pitavastatin, for which a clinical-event benefit has been demonstrated in an appropriately powered trial.

The cystatin C and microalbuminuria findings in the pidotimod pilot should therefore be interpreted as physiological shifts of uncertain durability. On their own, they do not justify a phase III trial powered for renal, cardiovascular, or mortality endpoints. A staged program is more appropriate: an independently replicated, adequately powered phase II trial should first confirm target engagement, durability, safety, and a prespecified biological effect; only a credible and reproducible signal, supported by event-rate estimates, would justify a phase III outcomes trial.

### 6.6 Consistency with guidelines

The NIH Guidelines for Adults and Adolescents with HIV reserve immune-based interventions aimed at residual activation for research settings (US Department of Health and Human Services, 2025). The EACS Guidelines similarly prioritize durable viral suppression, vaccination, prevention, and evidence-based management of comorbidities and do not recommend probiotics or pidotimod as standard treatment for residual immune activation (Ambrosioni et al., 2026). This position reflects an unresolved translational gap: immune-activation biomarkers are prognostic, but their treatment-mediated reduction has not been validated as a surrogate for improved clinical outcomes. Pidotimod should therefore remain investigational for this indication.

## 7 Proposal for future research on pidotimod in HIV

A future multicenter, randomized, double-blind, placebo-controlled trial should define one principal therapeutic objective. An appropriate proof-of-concept primary endpoint would be a prespecified change in a limited immune-activation measure, such as sCD14 or IL-6, over 12–24 weeks, because the existing HIV signal is biological rather than clinical. Such a trial would test target engagement, not establish prevention of clinical events. A subsequent, larger pragmatic trial would be required to evaluate clinically documented infections or non-AIDS morbidity. CD4/CD8 recovery could be a secondary endpoint in an immunological non-responder subgroup.

Exploratory biomarkers may include D-dimer, hsCRP, salivary IgA, intestinal permeability markers, cystatin C, albuminuria, microbiome, and metabolome measures. They should be explicitly separated from validated clinical outcomes. A statistical analysis plan should prespecify the primary comparison, adjust for baseline values and stratification factors, control the false-discovery rate or use a hierarchical testing strategy for multiplex biomarkers, and report effect sizes with confidence intervals. Replication, longer follow-up, blinded outcome adjudication, and systematic recording of ART, antibiotics, vaccination, corticosteroids, and probiotic exposure would be essential.

Trained immunity should be tested only as an exploratory mechanistic program within such trials. Prespecified assays should evaluate whether any persistent response after pidotimod exposure is accompanied by reproducible functional rechallenge, chromatin-accessibility or histone-mark changes, transcriptional remodeling, and metabolic reprogramming.

## 8 Conclusions

Selected probiotic formulations and pidotimod act through different biological routes and are considered together here only within a complementary, hypothesis-generating gut-immune framework. Probiotic findings in treated HIV are strain-, formulation-, dose-, population-, and endpoint-specific; they do not establish a generalized probiotic class effect. Pidotimod has mechanistic plausibility and preliminary adult signals in respiratory infection and HIV, but the supporting studies are generally small and heterogeneous.

Pidotimod therefore remains investigational for residual immune activation in treated HIV. Adequately powered, independently replicated randomized trials should distinguish immune restoration, control of pathological activation, and enhancement of host defense; prespecify a single primary objective; and separate exploratory biomarkers from clinically meaningful outcomes.
